# One Anastomosis Gastric Bypass Versus Long Biliopancreatic Limb Roux-en-Y Gastric Bypass

**DOI:** 10.1007/s11695-021-05874-0

**Published:** 2022-01-11

**Authors:** Mohamed Y. Ibrahim, Abdelmoneim S. Elshennawy, Arsany Talaat Saber Wassef, Ayman Salah, Ahmed M. Hassan, Sameh Mikhail

**Affiliations:** Department of General Surgery, Faculty of Medicine, Cairo University Hospitals Manial, Cairo, 11555 Egypt

## Abstract

**Background:**

Roux-en-Y gastric bypass (RYGB) is one of the most effective bariatric procedures. The study aimed to explore the value of lengthening the biliopancreatic limb (BPL) in RYGB compared to the outcome of one-anastomosis gastric bypass (OAGB).

**Methods:**

This prospective study included morbidly obese patients divided into two groups. The RYGB group (*n* = 36) was subjected to long biliary limb Roux-en-Y gastric bypass (LPRYGB), and the OAGB Group (*n* = 36) had one anastomosis gastric bypass. During follow-up, weight, BMI, percentage of excess body weight loss (%EBWL), resolution of obesity-related comorbidities, and quality of life (QoL) were evaluated.

**Results:**

There was no significant difference in weight and BMI after 3 and 6 months. At 12-month follow-up, weight loss was significantly higher in the OAGB group. After 12 months, the two groups showed significant improvement of comorbid conditions without significant difference between the two groups. The Qol was significantly higher in the LPRYGB group 3, 6, and 12 months after surgery compared to the OAGB group.

**Conclusions:**

Extending the BPL length in RYGB to 150 cm is as effective as OAGB in remission of comorbidities, including diabetes. It was also equally effective in weight reduction in the short term. OAGB was more efficient in weight reduction and a significantly faster operation. LPRYGB showed a better QoL of life 1 year after surgery.

**Graphical abstract:**

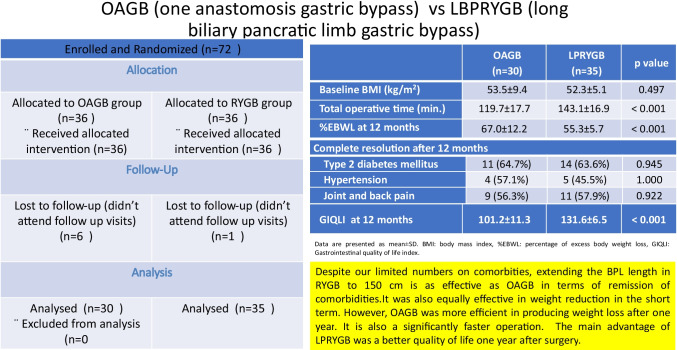

## Introduction

The prevalence of obesity continues to rise unrelentingly. Its prevalence varies by country ranging from 3.7 to 38.2% [1]. Bariatric surgery is the most effective long-term treatment for morbid obesity and associated type 2 diabetes (T2DM) [2]. Roux-en-Y gastric bypass (RYGB) has been one of the most effective bariatric procedures for the last five decades [3].

Many technical elements of RYGB have undergone variations, including the formation of the gastric pouch, staplers or hand-sewn anastomosis, and limb lengths [4]. So far, there is no consensus on the ideal limb lengths. In a large survey, the biliopancreatic limb (BPL) and alimentary limb (AL) lengths broadly varied from 10 to 250 and 35 to 250 cm, respectively [5]. According to a review article, the AL length is usually 100–150 cm, and the BPL length is usually 50 cm [6].

A recent systematic review of 13 articles concluded that weight loss is superior after gastric bypass surgery with longer biliopancreatic limbs [7]. Besides, gastric bypass with longer BPL has been associated with a high remission rate of diabetes [8]. OAGB is mounting steadily in popularity as a more straightforward, safe, and effective procedure compared to RYGB [9]. Its long-term outcome appears to be better concerning weight loss and diabetes control [10].

Therefore, the current study was designed to explore whether lengthening the biliopancreatic limb in RYGB can produce similar results to OAGB in terms of initial weight loss, resolution of obesity-related comorbidities, and quality of life.

## Patients and Methods

This prospective study included 72 morbidly obese patients seeking weight loss surgery in Cairo University Hospitals. We proposed that OAGB will achieve a weight loss higher than RYGB with longer BPL by 10% with a standard deviation of 12%. Based on these assumptions, 31 subjects in each group will be needed to be able to reject the null hypothesis that the population means of the two groups are equal with a power of 0.9 and an alpha error of 0.05. The sample will be raised by 15% to compensate for loss to follow up. Therefore, 36 patients in each group were included. The sample size was estimated using the G*Power© software (Institutfür Experimentelle Psychologie, Heinrich Heine Universität, Düsseldorf, Germany) version 3.1.9.2. They were randomized into two groups according to the type of bariatric procedure using computer-generated table. RYGB group (*n* = 36) were subjected to long biliary limb Roux-en-Y gastric bypass (LPRYGB). Patients in the OAGB Group (*n* = 36) had one anastomosis gastric bypass. Written informed consent was obtained from each patient, including an explanation of the procedure, description of the technique, the possible side effects, and outcome which may be favorable or not.

Inclusion criteria were morbidly obese patients 18 to 60 years old with acceptable operative risks. All patients should have failed an adequate conservative program (diet, exercise, and/or medication) for at least 6 months and were able to comply with nutritional supplementation and long-term follow-up. Morbid obesity was defined as a body mass index (BMI) > 40 kg/m^2^ or > 35 kg/m^2^ with obesity-related comorbidities.

Previous open abdominal surgery related to the gastrointestinal tract (GIT), including revision bariatric surgery, endocrine disorders other than diabetes mellitus and thyroid disorders, pregnancy or lactation, psychiatric illness, and a recent diagnosis of malignancy, were the exclusion criteria.

### Preoperative Assessment

All participants were subjected to full history taking and clinical examination with the calculation of BMI, ideal body weight, and excess body weight. Ideal body weight was calculated as height in meters squared (m^2^) multiplied by 25. Excess body weight = baseline weight − ideal body weight. Preoperative laboratory investigations included fasting blood glucose (FBG), kidney and liver function tests, coagulation and lipid profile, and serum iron calcium and magnesium. Other investigations included abdominal ultrasound, upper gastrointestinal endoscopy, ECG, and echocardiography if needed.

### Preoperative Preparation

Low-calorie (800–1000 kcal/day) pure high-protein diet with micronutrients and vitamins were administered for two weeks. Antibiotic prophylaxis in the form of an intravenous injection of third-generation cephalosporin was administered two hours before surgery. Low molecular weight heparin (LMWH) (40 iu subcutaneous) 12 h before surgery was used for thrombo-prophylaxis.

### Surgical Technique

The patient was placed supine with the operating table inclined to maximum reverse Trendelenburg. A carbon dioxide (CO_2_) pneumoperitoneum was established to 15-mmHg pressure using a veress needle. Direct optical entry to the abdominal cavity was carried out under vision using a 0-degree laparoscope. This laparoscope was then changed to a 30-degree scope. Five ports were placed in a “diamond-shaped” pattern in the upper abdomen: (1) 10-mm camera port, in the midline approximately two handbreadths below the xiphisternum; (2) 10-mm liver retractor port; (3) 12-mm right hand working port, in the left midclavicular line; (4) 12-mm left hand working port, in the right midclavicular; and (5) 5-mm assistant port, in the left anterior axillary line.

#### Constructing the Gastric Tube

For those having LPRYGB: the gastric pouch is about 8 cm long and was constructed snug on the bougie. The left-hand working port fired the first endoscopic stapler loaded with a 45-mm (blue) cartridge perpendicular to the lesser curvature. A 36-Fr bougie was advanced under direct vision to calibrate the gastric reservoir. Fatty tissue and fibrous adhesions between the posterior gastric wall and pancreas were dissected. Then, an endoscopic stapler loaded with 60-mm (blue or gold) cartridges was consecutively applied parallel to the lesser curvature, sectioning the stomach vertically, completing the gastric reservoir. In OAGB the gastric pouch is about 20–24 cm as long as we can.

#### Fashioning Anastomoses

Attention is turned to the left gutter, ligament of Treitz is identified, and unstretched small bowels are measured along the ante-mesenteric border. OAGB was completed by creating an ante-colic gastro-jejunostomy (GJ) 200 cm from DuodenoJeujenal flexure. Petersen’s defect is not closed. For those having LPRYGB: 150-cm BPL was measured. GJ was performed in the same technique of OAGB (using a 45-mm blue stapler). Side to side stapled jejuno-jejunostomy (using a white cartridge) was performed 60 cm from GJ. In both anastomoses, enterotomies were closed using a hand-sewn running 3/0 Vicryl stitch in 2 layers. Roux-en-Y reconstruction was completed by dividing the afferent limb using a 60-mm stapler. The integrity of the GJ was assessed by Methylene blue test. Both Petersen’s and mesenteric defects are then closed. Staple line bleeding was controlled. A drain was inserted in some cases.

#### Postoperative Management

All patients were given a 3^rd^ generation cephalosporin, low molecular weight heparin (LMWH), opioid analgesia, proton pump inhibitors (PPIs), and antiemetics. Early postoperative ambulation was encouraged. Oral clear fluids were started 4 to 6 h postoperatively. On postoperative day (POD) one, the abdominal drain (if present) was removed, and the patient was discharged home as long as there were no complications. During the first 2 weeks, all patients were placed on a liquid-only diet. This was then advanced to a mashed food for two weeks, followed by semi-solid diet for another two weeks. After that, a regular healthy diet was started. A standardized supplementation regime was prescribed for life, including daily pills of calcium citrate and multivitamins containing vitamins A, E, C, B1, B2, B6, B12, and D, folic acid, phosphorus, iodine, iron, magnesium, manganese, potassium, chlorine, zinc, and nickel.

Postoperative follow-up visits were scheduled 1 week and 1 month after surgery to exclude early postoperative complications and then at 3, 6, and 12 months after surgery to monitor weight loss, quality of life (QoL), and resolution of obesity-related comorbidities. In between these timeframes, we kept close contact through telephone.

At each follow-up visit, clinical evaluation included Actual weight and BMI, percentage of excess body weight loss (%EBWL), resolution of obesity-related comorbidities, evaluation for nutritional or vitamins deficiency. The %EBWL was calculated as follows: [(baseline weight − actual weight)/(baseline weight − ideal bodyweight)] × 100.

Quality of life (QoL) was measured by the Gastrointestinal Quality of Life Index (GIQLI) [11]: It is a 36-item questionnaire, each item is quoted 0–4. The questionnaire measures five principal domains: upper gastrointestinal symptoms (12 items), lower gastrointestinal symptoms (7 items), physical status (7 items), psychological status (5 items), and social status (5 items). The scores range from 0 to 144, with higher scores indicating better function.

Diabetes remission is defined as achieving glycemia below the diabetes range in the absence of active pharmacological or surgical therapy. Partial remission was defined as subdiabetic hyperglycemia (HbA1c < 6.5% and fasting glucose 100–125 mg/dL) for at least 1 year, and complete remission is a complete return to normal glucose metabolism measurements (normal HbA1c and fasting glucose < 100 mg/dL) for the same duration [12].

Hypertension remission, defined as systolic and diastolic blood pressure < 140 and 90 mmHg, respectively, with previous withdrawal of all medication [13].

We relied only on symptomatic improvement for assessment of back/joint pain resolution.

### Statistical Methods

Data were analyzed using SPSS version 26 (IBM Corp., Armonk, NY, USA). Data were summarized using mean and standard deviation for quantitative variables and frequencies and percentages for categorical variables. Comparisons between groups were made using unpaired *t*-test for numerical variables and Chi-square test (Fisher’s exact test) for categorical variables. A *p*-value < 0.05 was considered significant.

## Results

**Fig. 1:**
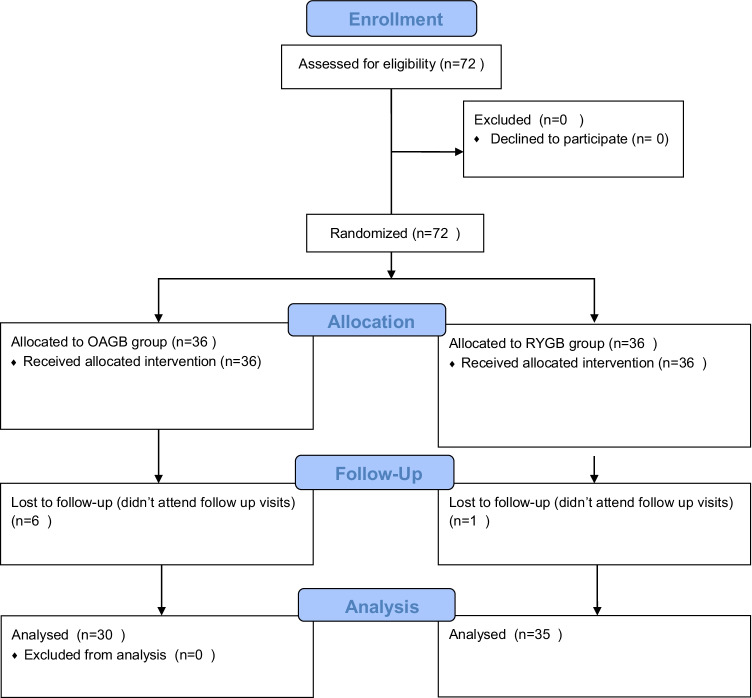
CONSORT 2010 Flow chart

Twelve months after surgery, one patient of LPRYGB and six of the OAGB group were lost to follow up. The results are presented for the remaining 65 patients (Fig. 1).

Table 1 summarizes the baseline characteristics of the two studied groups. The two groups were comparable in age, weight, height, and BMI. More males were subjected to LPRYGB (*p* = 0.017), which was more lengthy operation (*p* < 0.001).

**Table 1:** Baseline characteristics and operative time of the two studied groups

	OAGB	LPRYGB	*p* value
	(*n* = 30)	(*n* = 35)	
Age (year)	37.7 ± 9.9	36.9 ± 10.2	0.728
Sex (female/male)	26/4	21/14	0.017
Height (cm)	164 ± 8.8	164 ± 8.2	0.777
Baseline weight (kg)	143.0 ± 25.8	141.5 ± 19.1	0.797
Baseline BMI (kg/m^2^)	53.5 ± 9.4	52.3 ± 5.1	0.497
Ideal body weight (kg)	67.1 ± 7.3	67.5 ± 6.8	0.833
Excess body weight (kg)	75.9 ± 24.5	73.9 ± 14.8	0.698
Total operative time (min)	119.7 ± 17.7	143.1 ± 16.9	< 0.001

Data are presented as mean ± SD

*BMI*, body mass index

There was no significant difference in weight and BMI after 3 and 6 months. At 12-month follow-up, weight loss was significantly higher in the OAGB group (Table 2). At baseline, there was no significant difference between the two groups in the proportion of patients with T2DM, hypertension, or joint and back pain. By the end of the follow-up period, the two groups had shown significant improvement of associated comorbid conditions in all patients. Complete resolution of T2DM, hypertension, and joint and back pain was encountered in half or more than half of cases in both groups without significant difference between them (Table 3). The GIQLI was significantly higher in the LPRYGB group 3, 6, and 12 months after surgery compared to the OAGB group (Table 4).

**Table 2:** Weight change and body mass index 3, 6, and 12 months after surgery in the two studied groups

	OAGB	LPRYGB	*p* value
	(*n* = 30)	(*n* = 35)	
After 3 months			
Weight (kg)	120.8 ± 22.3	120.6 ± 16.0	0.972
BMI (kg/m^2^)	45.2 ± 8.2	44.4 ± 4.3	0.593
Excess weight loss (kg)	22.2 ± 6.5	20.9 ± 3.5	0.319
%EBWL	31.0 ± 10.6	28.6 ± 4.1	0.216
After 6 months			
Weight (kg)	108.5 ± 19.1	109.0 ± 14.2	0.919
BMI (kg/m^2^)	40.6 ± 7.1	40.1 ± 3.8	0.711
Excess weight loss (kg)	34.5 ± 9.4	32.6 ± 5.0	0.313
%EBWL	47.5 ± 12.8	44.5 ± 4.9	0.214
After 12 months			
Weight (kg)	92.9 ± 13.3	100.9 ± 13.0	0.017
BMI (kg/m^2^)	34.8 ± 4.8	37.2 ± 3.5	0.024
Excess weight loss (kg)	50.1 ± 16.0	40.6 ± 6.5	0.002
%EBWL	67.0 ± 12.2	55.3 ± 5.7	< 0.001

Data are presented as mean ± SD

*BMI*, body mass index, *%EBWL*, percentage of excess body weight loss

**Table 3:** Resolution of associated comorbidities in the two studied groups

	OAGB	LPRYGB	*p* value
	(*n* = 30)	(*n* = 35)	
Comorbid conditions at baseline			
Type 2 diabetes mellitus	17 (56.7%)	22 (62.9%)	0.612
Hypertension	7 (23.3%)	11 (31.4%)	0.467
Joint and back pain	16 (53.3%)	19 (54.3%)	0.939
Complete resolution after 12 months			
Type 2 diabetes mellitus	11 (64.7%)	14 (63.6%)	0.945
Hypertension	4 (57.1%)	5 (45.5%)	1.000
Joint and back pain	9 (56.3%)	11 (57.9%)	0.922

Data are presented as number (%)

**Table 4:** Postoperative quality of life in the two studied groups measured by Gastrointestinal Quality of Life Index

	OAGB	LPRYGB	p value
	(*n* = 30)	(*n* = 35)	
After 3 months	91.8 ± 11.2	97.1 ± 5.6	0.015
After 6 months	98.3 ± 12.3	123.1 ± 11.6	< 0.001
After 12 months	101.2 ± 11.3	131.6 ± 6.5	< 0.001

Data are presented as mean ± SD

## Discussion

This study aimed to explore if lengthening the BPL in RYGB can attain a better outcome in morbidly obese patients. We compared the effect of this modified RYGB with OAGB, which has been shown to be superior in weight loss and remission of comorbidities. The results demonstrated that a longer BPL in RYGB is as good as OAGB in controlling comorbid conditions, including T2DM, and producing short-term weight loss. But, after 12 months of surgery, weight loss was significantly higher in the OAGB. LPRYGB had the advantage of a significantly higher quality of life compared to OAGB.

Many studies demonstrated the superiority of OAGB to RYGB. A recent meta-analysis of 11 studies involving 12,445 patients confirmed that OAGB is associated with more weight loss up to 5 years postoperatively and superior remission rates of T2DM compared to RYGB [14]. This superiority could be attributed to its more malabsorptive traits [14]. We believe that a longer BPL could increase the malabsorptive component of RYGB to approach the better results of OAGB.

This notion was motivated by previous studies investigating the effect of elongation of the BPL in RYGB. Nergaard et al. compared the standard RYGB (AL: 150 cm, BPL: 60 cm) to diverted-OAGB with 60-cm AL and 200 BPL. The long BPL achieved significantly higher BMI loss after seven years than long AL (78.4% vs. 67.1%, respectively) [15]. Darabi et al. studied three variations of AL and BPL in RYGB (BPL 50 cm, AL 150 cm; BPL: 150 cm, AL: 50 cm; and BPL: 100 cm, AL: 100 cm). They found no difference in %EWL after 1 year. However, longer BPL achieved a higher %EWL after 3 years [16]. Another RCT compared 150-cm BPL, AL 75 cm, with BPL 75 cm, AL 150 cm. They found a significantly higher %EWL in cases of long BPL RYGB 4 years after surgery [17]. A BPL of 200 cm and AL of 50 cm were proved effective in achieving acceptable weight loss and remission of T2DM [18]. In diabetic patients subjected to RYGB, a longer BL of 100–150 cm had a better antidiabetic effect compared to the standard length of 50–75 cm [19].

In the current study, we used the reversed ratio of AL and BPL by a longer BPL (150 cm) with a shorter AL (60 cm). Generally, systematic reviews concluded that the combined length of the two limbs between 100 and 200 cm might yield optimal results in proximal bypass [20].

Besides, in failed laparoscopic sleeve gastrectomy cases, revisional surgery with long BPL procedures (RYGB or OAGB) ensued a significant long-term weight loss at three years [21]. A similar benefit was reported in revisional surgery after failed RYGB. A long BPL RYGB was associated with significantly higher %EWL and TWL at five years postoperatively [22].

However, the mechanism underlying better weight loss with long BPL in RYGB remains uncertain. It may lead to superior stimulation of the distal intestine and altered bile acids and intestinal microbiota [18]. Also, available evidence indicates that long BPL in biliopancreatic diversion can be a key factor in explaining this procedure’s superiority in achieving weight loss [21]. A longer BPL bypassed more of the jejunum, leading to early malabsorption of nutrients, causing a significant early loss of weight. However, in the long term, the weight loss effect decreased. This is confirmed in the current study, where weight loss was comparable to OAGB up to 6 months, but it was significantly lower after one year in LPRYGB. The bariatric procedure generally generates mild fat malabsorption due to many factors, including an inadequate mixing with digestive secretions [23, 24]. The passage of food directly to the ileum could affect food tolerance and, consequently, eating behavior. With a long BPL, bypassing most of the foregut is might alter the hormonal and immunological factors. The difference in the GI hormones’ profile demonstrated in recent studies may be the primary mechanism [25].

In fact, variable BPL lengths were investigated in OAGB. A retrospective analysis compared the outcome of OAGB with BPL of 150 cm, 180 cm, and 250 cm. Nutritional deficiencies were more common in the 250-cm group compared to the 150-cm group. The difference between 150 cm vs. 180 cm was insignificant regarding weight loss and resolution of T2DM and hypertension [26]. Komaei et al. [27] suggested that tailoring BPL length by bypassing about 40% of the small bowel length is safe and effective and appears to be superior to the fixed 200-cm BPL. We believe that 200 cm is within 40% of the small bowel length of most patients. Lee et al. suggested tailoring BPL lengths to patients’ preoperative BMI to be 150 cm for BMI > 40 kg/m^2^, 250 cm for BMI 40–50 kg/m^2^, and 350 cm for BMI > 50 kg/m^2^. Higher BMI reduction was associated with longer BPL [28].

In the current study, the AL length was 60 cm. A recent meta-analysis concluded that in patients with a BMI < 50, a relatively short AL of 40–100 cm is as useful as longer lengths (130–150 cm) in terms of weight loss [29]. Actually, older studies suggested that a long AL of 150 cm and lengthening the BPL to 30 cm was associated with greater weight loss at 24 and 36 months. However, three more recent systematic reviews concluded that a longer AL does not significantly impact patients with a BMI < 50 [20, 30, 31].

Our study included a relatively small number of patients followed up only for one year. Nutritional results at 1 year were not presented. Data of trace elements and vitamin deficiency at 1 year is incomplete as some patients couldn’t afford them. COVID-related restrictions made follow up at 1 year mainly by phone. Our results did not segregate patients who had complete remission of their comorbidities from those who had partial remission.

We can conclude, despite our limited numbers on comorbidities, that extending the BPL length in RYGB to 150 cm with an AL of 60 cm is as effective as OAGB in terms of remission of comorbidities, including diabetes. It was also equally effective in weight reduction in the short term. However, OAGB was more efficient in producing weight loss after 1 year. It is also a significantly faster operation. The main advantage of LPRYGB was a better quality of life 1 year after surgery. Research of the appropriate length of functioning small bowel in gastric bypass surgery remains to be determined in large RCTs.

## References

[1] NCD Risk Factor Collaboration (NCD-RisC). Worldwide trends in body-mass index, underweight, overweight, and obesity from 1975 to 2016: a pooled analysis of 2416 population-based measurement studies in 128·9 million children, adolescents, and adults. Lancet. 2017;390:2627–42. 10.1016/S0140-6736(17)32129-3 PMC5735219 29029897

[2] Arterburn DE, Telem DA, Kushner RF, Courcoulas AP (2020). Benefits and risks of bariatric surgery in adults: a review. JAMA.

[3] Ahmed B, King WC, Gourash W, Hinerman A, Belle SH, Pomp A (2019). Proximal Roux-en-Y gastric bypass: addressing the myth of limb length. Surgery.

[4] Olbers T, Lönroth H, Fagevik-Olsén M, Lundell L (2003). Laparoscopic gastric bypass: development of technique, respiratory function, and long-term outcome. Obes Surg.

[5] Madan AK, Harper JL, Tichansky DS. Techniques of laparoscopic gastric bypass: on-line survey of American Society for Bariatric Surgery practicing surgeons. Surg Obes Relat Dis. 2008;4:166–72; discussion 172–173. 10.1016/j.soard.2007.08.006 18069071

[6] Abdeen G, le Roux C (2016). Mechanism underlying the weight loss and complications of Roux-en-Y gastric bypass. Review Obes Surg.

[7] Zorrilla-Nunez LF, Campbell A, Giambartolomei G, Lo Menzo E, Szomstein S, Rosenthal RJ (2019). The importance of the biliopancreatic limb length in gastric bypass: a systematic review. Surg Obes Relat Dis.

[8] Nora M, Guimarães M, Almeida R, Martins P, Gonçalves G, Freire MJ (2011). Metabolic laparoscopic gastric bypass for obese patients with type 2 diabetes. Obes Surg.

[9] Mahawar KK, Himpens J, Shikora SA, Chevallier JM, Lakdawala M, de Luca M, et al. The first consensus statement on one anastomosis/mini gastric bypass (OAGB/MGB) using a modified Delphi approach. Obes Surg. 2018;28(2). 10.1007/s11695-017-3070-2 29243145

[10] Musella M, Milone M, Deitel M, Kular KS, Rutledge R (2016). What a Mini/One Anastomosis Gastric Bypass (MGB/OAGB) is. Obes Surg.

[11] Eypasch E, Williams JI, Wood-Dauphinee S, Ure BM, Schmülling C, Neugebauer E (1995). Gastrointestinal Quality of Life Index: development, validation and application of a new instrument. Br J Surg.

[12] Buse JB, Caprio S, Cefalu WT, et al. How do we define cure of diabetes? Diabetes Care.2009;32 10.2337/dc09-9036 PMC2768219 19875608

[13] Benaiges D, Climent E, Goday A, et al. Bariatric surgery and hypertension: implications and perspectives after the GATEWAY randomized trial. Cardiovasc Diagn Ther. 2019;9. 10.21037/cdt.2018.10.04 PMC6382659 30881887

[14] Magouliotis DE, Tasiopoulou VS, Tzovaras G (2019). One anastomosis gastric bypass versus Roux-en-Y gastric bypass for morbid obesity: an updated meta-analysis. Obes Surg.

[15] Nergaard BJ, Leifsson BG, Hedenbro J, Gislason H (2014). Gastric bypass with long alimentary limb or long pancreato-biliary limb—long-term results on weight loss, resolution of co-morbidities and metabolic parameters. Obes Surg.

[16] Darabi S, Pazouki A, Hosseini-Baharanchi FS, Kabir A, Kermansaravi M (2020). The role of alimentary and biliopancreatic limb length in outcomes of Roux-en-Y gastric bypass. Wideochir Inne Tech Maloinwazyjne.

[17] Homan J, Boerboom A, Aarts E, Dogan K, van Laarhoven C, Janssen I (2018). A longer biliopancreatic limb in Roux-en-Y gastric bypass improves weight loss in the first years after surgery: results of a randomized controlled trial. Obes Surg.

[18] Murad AJ, Cohen RV, de Godoy EP, Scheibe CL, Campelo GP, Ramos AC (2018). A prospective single-arm trial of modified long biliopancreatic and short alimentary limbs Roux-en-Y gastric bypass in type 2 diabetes patients with mild obesity. Obes Surg.

[19] Kaska L, Kobiela J, Proczko M, Stefaniak T, Sledziński Z (2014). Does the length of the biliary limb influence medium-term laboratory remission of type 2 diabetes mellitus after Roux-en-Y gastric bypass in morbidly obese patients?. Wideochir Inne Tech Maloinwazyjne.

[20] Mahawar KK, Kumar P, Parmar C, Graham Y, Carr WRJ, Jennings N (2016). Small bowel limb lengths and Roux-en-Y gastric bypass: a systematic review. Obes Surg.

[21] Kraljević M, Süsstrunk J, Köstler T, Lazaridis II, Zingg U, Delko T (2021). Short or long biliopancreatic limb bypass as a secondary procedure after failed laparoscopic sleeve gastrectomy. Obes Surg.

[22] Kraljević M, Köstler T, Süsstrunk J, Lazaridis II, Taheri A, Zingg U (2020). Revisional surgery for insufficient loss or regain of weight after Roux-en-Y gastric bypass: biliopancreatic limb length matters. Obes Surg.

[23] Nuzzo A, Czernichow S, Hertig A, Ledoux S, Poghosyan T, Quilliot D (2021). Prevention and treatment of nutritional complications after bariatric surgery. Lancet Gastroenterol Hepatol.

[24] Lupoli R, Lembo E, Saldalamacchia G, Avola CK, Angrisani L, Capaldo B (2017). Bariatric surgery and long-term nutritional issues. World J Diabetes.

[25] Ciovica R, Takata M, Vittinghoff E, Lin F, Posselt AM, Rabl C (2008). The impact of roux limb length on weight loss after gastric bypass. Obes Surg.

[26] Ahuja A, Tantia O, Goyal G, Chaudhuri T, Khanna S, Poddar A (2018). MGB-OAGB: effect of biliopancreatic limb length on nutritional deficiency, weight loss, and comorbidity resolution. Obes Surg.

[27] Komaei I, Sarra F, Lazzara C, Ammendola M, Memeo R, Sammarco G (2019). One anastomosis gastric bypass-mini gastric bypass with tailored biliopancreatic limb length formula relative to small bowel length: preliminary results. Obes Surg.

[28] Lee W-J, Wang W, Lee Y-C, Huang M-T, Ser K-H, Chen J-C (2008). Laparoscopic mini-gastric bypass: experience with tailored bypass limb according to body weight. Obes Surg.

[29] Gan J, Wang Y, Zhou X (2018). Whether a short or long alimentary limb influences weight loss in gastric bypass: a systematic review and meta-analysis. Obes Surg.

[30] Orci L, Chilcott M, Huber O (2011). Short versus long Roux-limb length in Roux-en-Y gastric bypass surgery for the treatment of morbid and super obesity: a systematic review of the literature. Obes Surg.

[31] Stefanidis D, Kuwada TS, Gersin KS (2011). The importance of the length of the limbs for gastric bypass patients–an evidence-based review. Obes Surg.

